# Culture and Psychopathology: New Perspectives on Research, Practice, and Clinical Training in a Globalized World

**DOI:** 10.3389/fpsyt.2018.00366

**Published:** 2018-08-10

**Authors:** Carla Moleiro

**Affiliations:** Instituto Universitário de Lisboa (ISCTE-IUL), CIS, Lisbon, Portugal

**Keywords:** psychopathology, culture, explanatory models, cultural competences, psychotherapy

## Abstract

The present paper discusses the role of culture in understanding and treating psychopathology. It describes new perspectives on the conceptualization of psychopathology and on the definition of culture, and how these are intertwined. The impacts of culture, explicit and implicit discrimination, and minority stress on mental health are reviewed, especially in the current era. Culturally-sensitive assessment practices in psychopathology are emphasized, including addressing the multiple cultural identities of the patient, the explanatory models of the experienced distress, specific psychosocial stressors and strengths, and the cultural features of the practitioner-patient relationship in the clinical encounter. The particular case of psychotherapy in working with culturally diverse patients is explored. Finally, mainstreaming of culture in research and clinical training in psychopathology is highlighted, acknowledging that each clinical interaction is a cultural one.

It has never been truer that cultural context has a prominent role in understanding and treating psychopathology. In a globalized world, it is currently widely recognized that it is the cultural context that defines (mal)adjustment of human behavior, which includes how people usually behave, think, feel, and relate in social interactions. It also shapes the threshold of distress, and the range and forms of its expressiveness that are acceptable and adaptive. As with overall health and illness, psychological suffering implies an understanding of a complex, multi-dimensional process of biopsychosocial variables, which is culturally situated. Similarly, most treatments or interventions in face of psychopathology require the recognition of their historical roots in specific cultural perspectives, as culture also shapes psychotherapy models (1) and patient care in psychiatry, influencing every moment and every process in patient narratives of their suffering (2). In addition, culture also determines how credible and/or acceptable are treatment types in the eyes of a patient and his/her family (3), and consequently treatment adherence. Thus, culture is a key, undeniable current perspective on psychopathology and, for many authors, it has moved to the forefront in the study of psychopathology (4), parallel to the emerging impact of social neuroscience (5).

## New perspectives on the definitions of psychopathology and of culture

The focus on culture when understanding psychopathology has not always been present and it is still not mainstreamed in clinical psychology and psychiatry. In fact, throughout most of its history, psychopathology has neglected to address cultural diversity, as health sciences have easily labeled behaviors, cognitions, emotional, and social functioning as psychopathological for their deviance from social norms—usually defined in a Western, Eurocentric perspective. An illustration of this perspective lies on the tradition of “deviant” or “abnormal psychology” in the literature - attempts to understand and control behavior deemed to be aberrant or deviant from a statistical, functional, or moral standard (6).

Moving away from more traditional conceptions of statistical and social norms as indicators of psychopathological functioning, main psychopathology classification systems (7, 8) currently focus on the role of subjective distress, dysfunction and impairment (6). In other words, the presence of clinically significant subjective distress that is experienced by the patient, and the experience of impairment to one or more of the patient's areas of functioning (i.e., social, occupational or educational functioning) are core elements for conceptualizing psychopathology [see (7)]. In the DSM-5, APA (7) conceptualizes mental disorders as those conditions with clinically significant dysfunction in the individual, underlined by patterns of functioning in cognition, emotional regulation, or behavior that are associated with distress or disability. APA (7) adds that (i) this pattern must not be merely an expected and culturally sanctioned response to a particular event, stressor or situation; and that (ii) deviant behavior (e.g., political, religious, sexual) in relation to society does not represent psychopathology in itself. This definition seeks to acknowledge the interaction of biological and psychological processes, and sociocultural systems.

Even after its expansion in the 80's [see (9)], efforts to mainstream cross-cultural psychiatry were not present until recent years, and its impact on clinical practice and training has been slow to observe. Nonetheless, in recent years we have witnessed a growth in the literature on social and cultural psychiatry, with increasing recognition of the influence of culture as a key factor in the prevalence, clinical manifestation, diagnosis, treatment response and outcomes of mental illnesses for individuals [see (2, 10, 11)]. This has also resulted in a quite novel perspective on the conception of culture itself, in light of critics of group-based definitions of culture (based usually on nationality or racial/ethnic background), mostly arising from social sciences (e.g., anthropology). Two key novel elements in the conception of culture ought to be highlighted. First, current definitions of culture in mental health research and practice acknowledge the role of multiple collective influences that combine to constitute a person's identity. These influences arise from diverse origins, not only nationality, migration status, racial and/or ethnic origin, language, religion, and spirituality, but also age, gender identity, sexual orientation, socioeconomic and educational class, and functional status. These influences overlap in unique or particular ways, resulting in specific experiences of a given individual or group, for instance, with impacts on interdependent systems of discrimination or disadvantage [i.e. intersectionality—(12, 13)]. In all, these multiple lenses influence how a patient views the world, how he/she experiences it emotionally, and how he/she behaves in relation to other people. Secondly, recent conceptualizations of culture regard it as processual (11). This process of meaning-making is dynamic and interpersonal, as those multiple facets of one's identity become more or less prominent at any given moment, in the presence of some social interactions and contexts, and not others. This includes the clinical encounter. This notion underlies the most recent revision of the DSM [DSM-5, (3)], which recognizes the importance of a cultural case formulation of any patient's presenting complaint and clinical history, and the understanding of culture as a process, rather than synonymous with static group membership.

## Explaining psychopathology: the role of culture, discrimination and minority stress on mental health

Culture has a recognized role in not only conceptualizing psychopathology, but also in explaining and accounting for experienced distress, health and illness (14, 15). Certain conditions surrounding minority stress [gender; sexual orientation; e.g., (16)] and migration processes [e.g., (17)] may increase vulnerability, and stigmatized groups may be exposed to a higher number of risk factors for psychological distress [e.g., related to legal status, perceived discrimination, social exclusion, stigmatization, and victimization; (18)]. For instance, lesbian, gay, bisexual and transgender (LGBT) populations have been found to present increased risk for suicide (19, 20), traumatic stress reactions (21), major depression disorders (22, 23), anxiety disorders (24, 25), among others [e.g., (26)]. Also, socio-economic adversities, including poverty and environmental risk factors, have been associated with the onset and maintenance of psychopathological symptoms and low life satisfaction (27). This relationship has been explained through material deprivation but also increased adverse life events (such as unemployment, abuse and neglect), with consequences for treatment outcomes, including among children and adolescents [e.g., (28)]. Given the recent recession period and current socio-economic strain for many individuals, it seems relevant to recognize that people living in poverty are more likely to experience mental health problems (29), less likely to access treatment (30) and less likely to achieve full recovery from emotional psychopathological problems (31).

In addition, contemporary migration has an unprecedented mobility with an estimated number of 232 million international migrants in the world [World Migration Report; (32)]. Forced migration, steadily increasing as a result of armed conflict (both within and between nations), but also political, economic, social, and climate changes, has most recently been discussed in the mental health field, with undeniable impacts on health and psychological functioning (33). The effects of pre-migration and migration-related trauma among refugees have been acknowledged and documented (34).

Recent literature has emphasized the role of not only explicit discrimination, but also implicit attitudes in interpersonal interactions [evaluations automatically activated by the actual or symbolic presence of a social object; (35–38); see Hall et al., (39) for a systematic review]. Micro-aggressions in daily life [continuous experiences of aggression, often invisible to the perpetrator, who unconsciously holds biases and prejudice; (40–42)] have also been investigated, and have been found to have significant effects on an individual's well-being.

In sum, culture and other related socio-contextual factors, such as minority stress, discrimination and exposure to interpersonal violence, influence the development of clinically significant distress and resulting disability.

## On culturally-sensitive assessment of psychopathology

Assessment bias [called “cultural malpractice” by Dana (43)] has been identified as an issue in a variety of measures of personality and psychopathology among individuals from diverse backgrounds. This construct and method bias has a variety of sources (e.g., including instrument development, standardized test norms that under-represent social minority groups, neglect for language barriers and acculturation processes), and permeate the assessment process and results, and treatment recommendations. For instance, the use of the Minnesota Multiphasic Personality Inventory (MMPI-2; one of the most widely used and researched psychodiagnostic self-report measures in the world) among diverse patients has raised concerns (related to conceptual, metric, and functional equivalence) as it may not be appropriate among those whose worldviews differ from the Euro-American culture (43).

In an effort to strengthen culturally-sensitive assessment practices, aligned with the DSM-5 (7), the Cultural Formulation Interview (3) was developed. This interview represents a proposal for cultural assessment for use in routine clinical care. It presents a conceptual framework for clinicians to identify the role of culture on the patient's clinical presentation and care, in four domains: (1) cultural identity of the individual; (2) cultural explanations of the experienced signs and symptoms (i.e., explanatory models of illness); (3) cultural factors that may be associated with the psychosocial environment and levels of functioning (i.e., protective and risks factors); and (4) cultural features involved in the communication and the clinical relationship between the patient and the psychiatrist or psychologist.

The concept of explanatory models of the experienced distress was introduced in psychiatry by Kleinman (10), highlighting the clinical relevance of eliciting the patient's understanding of his/her own symptoms. These models stressed the predictive value of the patient representations of his/her illness (causes of the problems and its effects over time on different realms of life) on coping and help-seeking behaviors, and consequently treatment adherence and outcome [see (44), for a review]. Addressing the patient's explanatory models may, hence, maximize engagement and adherence; improve therapeutic alliance; strengthen empathy and positive expectations, while decreasing stigma, shame and other catastrophic beliefs [i.e., “weakness,” “going mad,” (3)].

Even though the evaluation of specific psychosocial stressors has been emphasized, a strength-based approach has also been pointed out as a valuable perspective on culturally-sensitive assessment among patients of stigmatized social groups (3). This includes the evaluation of the social network of the patient (e.g., extended family, migrant and religious communities, LGBT associations), as it may play a pivotal role in both the onset and development of psychopathology, as well as a buffer of the effects of risk or stressful factors and the course and outcomes of mental health conditions (45).

In light of the aforementioned definition of culture, cultural identity is conceptualized in a intersectional perspective, and encompasses (i) aspects related to national, ethnic, and racial background, including language and migration, as well as social economic and educational status; (ii) spirituality, religion, and moral traditions; and (iii) gender, gender identity, and sexual orientation. Hence, a particular example of an important aspect to assess is patient's religiosity and spirituality. Indeed, religion and spirituality represent key dimensions when aiming a complete understanding of an individual [e.g., (46)], as well as having a potential positive impact on both physical and mental health. Purpose, meaning making and connection to others and the transcendent (through religion and spirituality) may influence one's core beliefs, emotions and behaviors (47). Religiosity and spirituality may have different impacts across one's life spam, on mental health outcomes and to the psychological treatment process, with some positive and some negative impacts (46–48). However, religious and spiritual dimensions have been separated from mental health care in the nineteenth century (49) and patients' spiritual experiences oftentimes labeled as “bizarre” and pathologized.

Another particular example of a key dimension to consider in culturally-sensitive assessment is related to gender roles, gender identity and sexual orientation, as culture clearly shapes the roles of women and men in a society, their expressions of distress, and their interpersonal relations (14, 50). Culture also determines the way diversity in gender identities and expressions are understood, as well as diversity in human sexuality (i.e., sexual orientation). In addition, mental health research has mainly treated sexual and ethnic identities separately, focusing on either of these two domains, with a few studies in the field addressing the experiences of individuals whose minority cultural/ethnic identities intersect with non-normative sexuality/gender expression [e.g., coping and resilience among Black lesbians; (51)].

In sum, culturally-sensitive psychopathology assessment will require the clinician to identify the cultural identities of the patient; conceptualizing his/her distress in a cultural lens; evaluate psychosocial stressors and protective factors; and be mindful of the cultural features of the relationship between the patient and the clinician and how the clinical encounter plays a role in the overall evaluation process (52).

## Psychotherapy: working with cultural diversity

Since the seminal work by Frank and Frank (53), psychotherapy has been compared with diverse healing practices or treatments across different times and different cultures (54). Still, in a globalized world, even though the need for culturally competent mental health services has been well recognized, health and mental health care disparities have been largely documented. While meta-analysis have shown a moderate effect size of culturally adapted interventions (55), studies in psychotherapies across many disorders have concluded that outcomes for minority cultural groups are not as good as for the majority populations and found greater rates of premature termination (1, 52). The sources of these disparities in healthcare are complex and exist in a broader historical and contemporary context of social and economic inequality, prejudice, and systematic bias (18). In fact, the Western biomedical health model has created a professional culture, based on specific values (e.g., power, agency, objectivity, individualism), which may differ from the diverse cultures of those attending health services. Psychotherapeutic theoretical models (e.g., psychoanalytic, psychodynamic, humanistic, cognitive-behavioral, systemic approaches) have also been historically rooted in concepts and developed in contexts that were not sensitive to the current cultural diversity. In other words, the healthcare system itself can be less in accordance with the cultural perspectives of some patients than others. Therefore, clinicians' sense of social responsibility and social justice concerns have arisen as a response to social inequalities in mental health care, and specific culturally-sensitive treatments have been developed [e.g., multicultural counseling; LGBT affirmative psychotherapies; (56)].

A recent special issue of the Journal of Contemporary Psychotherapy critically reviewed the practice and development of psychotherapy in Nigeria (57), China (58), India (59), Saudi Arabia (60), Pakistan (61), and Israel (62). Iwakabe (63) had already done so for the Japanese context. These authors discuss the relevance and applicability of “Western” psychotherapies in different populations, considering distinct cultural, religious, political, social, familial, and individual features, with implications not only for treatment outcomes, but also the clinical therapeutic relationship. Another relevant, recent example is a special issue of Counseling and Psychotherapy Research (27), which brings light to the role of social inequalities in psychotherapy research and practice, acknowledging that, for a long time, psychotherapy was seen as an endeavor for the middle- and upper-class of educated and psychologically minded clients.

Moreover, only recently the impact of cultural diversity on practitioner-patient interactions has been examined, for instance in medicine (64). However, the American Institutes for Research had already acknowledged in 2002 that “*social issues such as stereotyping, institutionalized racism, and dominant-group privilege are as real in the examining room as they are in society at large*” [(65), p. 8]. That is, issues of stereotyping and discrimination may be as real in the clinician-patient relationship as in any other interpersonal relationship. Indeed, social, educational and economic disparities between patients and clinicians are often evident (27). Moreover, there is evidence of stereotyping and bias among healthcare providers [e.g., (39, 66, 67)], and diverse micro-aggressions in the health care systems (42). This is due to the fact that, even though negative explicit attitudes toward stigmatized groups have been declining, substantial implicit negative attitudes still exist and exert influence on behavior, from everyday encounters to clinical interactions (64). Still, blatant examples persist in the practices of many clinicians in helping patients redirect or change same-sex sexual orientation (68).

## Mainstreaming culture in research on psychopathology and in clinical training

The present paper argues for mainstreaming culture in research and clinical training in psychopathology, acknowledging that each clinical interaction is a cultural one. As aforementioned, different characteristics of one's identity are salient in different contexts and interactions. In the practitioner-patient interaction, this is no exception and thus clinicians need to be able to be responsive to this cultural encounter—i.e., to be culturally competent.

Cultural competence is generally defined in a tri-dimensional model, as the extent to which clinicians possess appropriate awareness, relevant knowledge, and practical skills in working with individuals from diverse cultural backgrounds (11, 15, 18). The first dimension—awareness—refers to the way the clinicians' attitudes, beliefs, values, assumptions, and self-awareness affect how they interact with those patients who are culturally different from themselves. It involves the exploration of the self as a cultural being, and of one's own cultural preconceptions. The second dimension—knowledge—relates to the informed understanding of cultures that are different from one's culture, including their histories, traditions, values, practices, and so forth. It also involves knowledge about such concepts and processes as cultural impacts on psychosocial development, acculturation models and acculturation stress, social minority stress and identity development, cultural communication styles in the helping relationship, perceived discrimination and socioeconomic adversity as risks factors for well-being, among others. Finally, an important third dimension consists in the ability to engage in effective and meaningful interactions with diverse individuals, including the development of a relationship, by integrating one's awareness and knowledge into practical skills in the clinical relation, assessment and intervention. Cultural competence has been proposed as a strategy to respond to diversity in contemporary societies and make health care services more accessible, acceptable and effective for diverse communities. Initially intended for work with migrants and ethnic minorities, cultural competence has been extended to include other forms of client diversity, such as age, gender, sexual orientation, gender identity, religion, social class, language, and ability status [e.g., (69, 70)]. It has been proposed as a developmental process, both at an individual (i.e., the clinician) and an organizational (i.e., healthcare unit) levels. Despite recent debates and criticisms [cultural safety, cultural sensitivity, cultural responsiveness, and cultural humility; (11)], developing cultural competence in psychopathology is a key process aligned with person-centered care, where patient narratives and meanings are shared and interpreted in the clinical encounter. However, research is still needed on the processes of implementation, clinical effectiveness, clinical communication, wider social impact, and outcomes of culturally competent services and interventions (11, 39). Indeed, Delgadillo (27) argues for a better integration of the literature on social inequalities, power imbalance, and cultural competence into clinical training programmes. The recent aforementioned understandings of culture and of psychopathology in a social and cultural context, rather than an (exclusively) intra-individual process, provide a possible route to develop these clinical competences. While some have reported training pilot studies and their evaluation, and guidelines have been proposed [e.g., (3, 17)], clinical training in individual and cultural diversity is still scarce and unsystematic, both in the educational/academic process and in professional development. Addressing this gap and mainstreaming cultural competence in clinical training seems to be a key future development if we are to enable clinicians to provide support and address mental health concerns in a diverse world.

## Author contributions

The author confirms being the sole contributor of this work and approved it for publication.

### Conflict of interest statement

The author declares that the research was conducted in the absence of any commercial or financial relationships that could be construed as a potential conflict of interest.
